# Antibacterial Potential of Essential Oils and Silver Nanoparticles against Multidrug-Resistant *Staphylococcus pseudintermedius* Isolates

**DOI:** 10.3390/pathogens13020156

**Published:** 2024-02-09

**Authors:** Gabriele Meroni, Giulia Laterza, Alexios Tsikopoulos, Konstantinos Tsikopoulos, Sara Vitalini, Barbara Scaglia, Marcello Iriti, Luigi Bonizzi, Piera Anna Martino, Alessio Soggiu

**Affiliations:** 1 One Health Unit, Department of Biomedical, Surgical and Dental Sciences, School of Medicine, University of Milan, Via Pascal 36, 20133 Milan, Italy; giulia.laterza@unimi.it (G.L.); sara.vitalini@unimi.it (S.V.); marcello.iriti@unimi.it (M.I.); luigi.bonizzi@unimi.it (L.B.); piera.martino@unimi.it (P.A.M.); alessio.soggiu@unimi.it (A.S.); 2 Department of Clinical and Community Sciences, School of Medicine, University of Milan, Via Celoria 22, 20133 Milan, Italy; 3 1st Department of Pharmacology, School of Medicine, Faculty of Health Sciences, Aristotle University of Thessaloniki, 54124 Thessaloniki, Greece; alextsikop@yahoo.gr (A.T.); kostastsikop@gmail.com (K.T.); 4 Department of Agricultural and Environmental Sciences, University of Milan, Via Celoria 2, 20133 Milan, Italy; barbara.scaglia@unimi.it

**Keywords:** MRSP, MDR, essential oil, silver nanoparticles

## Abstract

*Staphylococcus pseudintermedius* is an emergent zoonotic agent associated with multidrug resistance (MDR). This work aimed to describe the antibacterial activity of four essential oils (EOs) and silver nanoparticles (AgNPs) against 15 *S. pseudintermedius* strains isolated from pyoderma. The four EOs, namely *Rosmarinus officinalis* (RO), *Juniperus communis* (GI), *Citrus sinensis* (AR), and *Abies alba* (AB), and AgNPs were used alone and in combination to determine the Minimum Inhibitory Concentration (MIC) and Minimum Bactericidal Concentration (MBC). All strains were MDR and methicillin-resistant. Among the antibiotic cohort, only rifampicin, doxycycline, and amikacin were effective. EOs’ chemical analysis revealed 124 compounds belonging to various chemical classes. Of them, 35 were found in AR, 75 in AB, 77 in GI, and 57 in RO. The monoterpenic fraction prevailed over the sesquiterpenic in all EOs. When EOs were tested alone, AB showed the lowest MIC followed by GI, AR, and RO (with values ranging from 1:128 to 1:2048). MBC increased in the following order: AB, AR, GI, and RO (with values ranging from 1:512 to 1:2048). MIC and MBC values for AgNPs were 10.74 mg/L ± 4.23 and 261.05 mg/L ± 172.74. In conclusion, EOs and AgNPs could limit the use of antibiotics or improve the efficacy of conventional therapies.

## 1. Introduction

Over the last decade, *Staphylococcus pseudintermedius* has gained attention due to its zoonotic potential, directly linked to the genetic acquisition of antibiotic-resistant genes (ARGs) and virulence factors [1,2,3]. Up to 97.8% of Methicillin-resistant. *pseudintermedius* (MRSP) isolates have shown multidrug resistance to three or more antibiotics commonly administered in veterinary medicine [4,5]. The colonization of *S. pseudintermedius* is quite similar to that of *S. aureus* in humans, with human nares being the most prevalent site of colonization, in contrast to the pharynx and rectum in companion animals (e.g., cats, dogs, horses) [1,6]. *S. pseudintermedius* is noted for its opportunistic potential, particularly in immunocompromised hosts, and is a typical commensal of dogs. Furthermore, it has been linked to several cases of human colonization and infection, most of which were caused by intimate contact between companion dogs and people [7,8,9,10]. *S. pseudintermedius* is more likely to adapt in humans due to the intimate interaction between companion animals (particularly dogs and cats), owners, and other individuals, such as small-animal veterinarians, as reported in the literature [11]. There are limited studies that discuss the transmission, colonization, and infection of humans by *S. pseudintermedius* because it is often misidentified as *S. aureus*. Additionally, most diagnostic laboratories cannot afford to use advanced technologies like MALDI-TOF MS, PCR with species-specific gene targeting, MLST, and whole-genome sequencing. Consequently, accurately determining the true occurrence of zoonotic transmission, human colonization, and infection, and the present epidemiology of this pathogen poses some difficulties [12].

As a result, the need for alternatives to standard antibiotics is urgent. In this context, secondary metabolites of plants including essential oils (EOs), produced in response to environmental conditions such as herbivore assault, abiotic stress, or interspecific interactions, have emerged as significant potential choices [13]. The use of EOs as antibiotic alternatives has attracted substantial attention in recent years, indicating a paradigm change in the approach to bacterial diseases management [14]. EOs are the primary components of aromatherapy, and they are produced by up to 17,000 distinct plant species from 60 different families (e.g., Lamiaceae, Rutaceae, Myrtaceae, Zingiberaceae, and Asteraceae) [15]. The wide and complicated chemical composition of EOs, which often comprises many bioactive substances functioning synergistically, is one of their primary benefits [16]. Because of this complication, it is difficult for bacteria to evolve specific resistance, which is a major concern with standard antibiotics [17]. Further researches are necessary to comprehensively comprehend the mechanisms of EOs and their suitable use in clinical settings [18,19]. In recent years, various in vivo and in vitro investigations have been conducted focusing on the efficiency of several EOs against the etiological agents of pyoderma in dogs [15,20,21,22]. Many EOs may be used to treat various skin disorders; however, due to their bioactive chemical components, some are particularly efficient against Gram-positive and Gram-negative bacteria [23], in particular those with significant percentages of thymol and carvacrol have extraordinary membrane-damaging action in bacteria [24,25,26].

A second rapidly-growing field is nanotechnology, aiming to synthesize and characterize nanoparticles (NPs) with several applications in different scientific disciplines [27]. As known, silver (Ag) is a noble metal used for centuries as an antibacterial agent and its chemical properties made it a valuable candidate as a metallic precursor for the synthesis of NPs [5]. Due to their unique physical and chemical characteristics, AgNPs demonstrated formidable antimicrobial and anti-inflammatory capabilities, making them interesting in dermatology [28,29]. Wound recovery is one of the principal applications of AgNPs [30]. Antimicrobial properties aid in the prevention of infections, while wound healing is expedited by their capacity to stimulate cell proliferation and collagen synthesis [31,32,33].

This study investigates the in vitro inhibitory (MIC) and bactericidal (MBC) effects of four commercially available EOs in conjunction with chemically-synthesized silver nanoparticles against multi-drug resistant *S. pseudintermedius*. For the best knowledge of the authors this is the first in vitro study focused on the combined use of NPs and EOs.

## 2. Materials and Methods

### 2.1. Bacterial Strains, Identification, and Culture Conditions

Fifteen *S. pseudintermedius* strains were chosen from the Department of Biomedical, Surgical, and Dental Sciences’ (University of Milan) bacterial collection. Bacteria were isolated starting from 201 from deep canine cutaneous pyoderma and stored at −20 °C in 25% (*v*/*v*) glycerol (Carlo Erba, Cornaredo, Italy). Original isolation was performed using both phenotypic and molecular techniques. In brief, phenotypic identification was made using Mannitol Salt Agar (Microbiol, Macchiareddu, Italy) as a selective and differential medium after the first isolation on Columbia Base Agar (ThermoFisher, Monza, Italy) with 5% defibrinated sheep blood (Microbiol, Italy). Molecular identification was conducted, [34] coupled with a Restriction Fragment Length Polymorphism (RFLP) assay [35]. After identification, each sample was stored in filtered (0.22 µm) glycerol at −20 °C. The day before the experiment, each sample was thawed at room temperature and plated on Columbia Base Agar (ThermoFisher, Italy) with 5% defibrinated sheep blood (Microbiol, Italy) and incubated aerobically at 37 °C for 18–24 h.

### 2.2. Determination of Antimicrobial Profiles

The Kirby–Bauer Disk diffusion assay was used to investigate the susceptibility of the strains to 22 antibiotic molecules following the Clinical and Laboratory Standard Institute guidelines [36]. The antimicrobials (abbreviations and concentrations in µg) used were Oxacillin (OX; 5), Amoxicillin + clavulanic acid (AMC; 30), Amoxicillin (AMO; 30), Carbenicillin (CAR; 100), Cephalexin (CL; 30), Cefovecin (CVN; 30), Ceftiofur (EFT; 30), Ceftriaxone (CRO 30, Clindamycin (DA; 10), Lincomycin + Spectinomycin (LC-SP; 15), Doxycycline (DO; 30), Enrofloxacin (ENR; 5), Marbofloxacin (MAR; 5), Pradofloxacin (PRA; 5), Amikacin (AK; 30), Gentamicin (CN; 30), Neomycin (N; 30), Tobramycin (TOB; 10), Kanamycin (K; 30), Rifampicin (RD; 30), Azithromycin (AZM; 15), Erythromycin (E; 30).

ARGs were detected using two sets of mPCR targeting *mecA*, *blaZ*, *aacA-aphD*, *tetM* and *tetK* genes, as previously described [15].

### 2.3. Synthesis and Characterization of Silver Nanoparticles

AgNPs were chemically synthesized using silver nitrate (AgNO_3_, Carlo Erba, Italy) as the metal ion donor, as described [37]. Briefly, a 100 mM solution of AgNO_3_ was prepared by dissolving 8.49 g of salt in 500 mL of distilled water and heated at 90 °C for 5 min. By dripping, 12.5 mL of 1% Tri-Sodium Citrate (TSC) in water was added. The reduction of metal ions was ascertained by the color change of the solution from transparent to brown. The volume was transferred into a separatory funnel and darkened for 24 h. After eluting, the volume fraction containing the NPs (approximately 50 mL) was centrifuged (4000 RCF, 15 min), washed twice with distilled water, and freeze-dried (CoolSafe Basic, Labogene, Scandinavia) for 24 h at −54 °C.

The reduction from Ag^+^ to Ag^0^ was monitored by measuring the ultraviolet-visible absorption spectrum UV-Vis (SpectraMax 340 PC, Molecular Devices (Germany) GmbH, Munich, Germany) at wavelengths from 310 to 770 nm (with a 50 nm path). Readings were taken twice within 15 min. Using a transmission electron microscope (EFTEM Leo 912ab (Zeiss, Milan, Italy)) at a voltage of 100 kV, the nanoparticles’ morphology was determined. Samples were briefly sonicated (30 kHz, 15 s on and 45 s off), and immediately, a drop of the aqueous AgNPs’ suspension was mounted on a carbon grid and placed on a filter paper to absorb excess solvent. The morphological analysis (diameter and particle size distribution) was calculated with ImageJ2 software (v. 1.54h).

### 2.4. Characterization of Essential Oils

The four EOs, *Rosmarinus officinalis* (RO), *Juniperus communis* (GI), *Citrus sinensis* (AR), and *Abies alba* (AB), were purchased from an Italian company (Vitalis, Milan, Italy).

The EOs were diluted in methanol (as reported in the literature [38]), and characterization was performed using an Agilent 5975C Series GC/MS and FID as the detector. Volatiles were separated using an apolar capillary column Zebron–Semivolatiles (Zebron, Phenomenex, Torrance, CA, USA) of 30 m × 0.25 mm (ID) and a film thickness of 0.25 µm. The carrier gas was helium at a flow rate of 1 mL/min. Two µLs of each sample were injected in the GC MS using a CTC PAL system in triplicates. The injector was set at 230 °C under splitless mode. The temperature program was isothermal for 3.5 min at 40 °C, then, two different temperature ramps were conducted to reach 140 °C, 150 °C (hold time of 7 min), and 220 °C (hold time of 23 min) at rates of 7 °C/min and 20 °C/min, respectively. The transfer line to the mass spectrometer was maintained at 150 °C. The mass spectra were obtained by electronic impact at 70 eV, a multiplier voltage of 1294 V, collecting data at a m/z range of 35–500. The retention indices were determined in relation to a homologous series of n-alkanes (C7–C30, Sigma Aldrich, Milan, Italy) under the same operational conditions. The chromatograms were elaborated using the open-source MS-DIAL using the NIST14 library as a reference, considering a total score (TS) = 70% as the cutoff of the data, as suggested by the Metabolomics Standards Initiative of the International Metabolomics Society [39]. The TS was a quality index applied in Ms-DIAL, calculated using (i) retention index similarity, (ii) accurate mass similarity, (iii) spectra similarity, and (iv) isotope spectra similarity of each compound [40]. The score of each parameter was standardized (0 = no quality and 1 = perfect match) and mathematically elaborated to yield a TS range of 0–100. For the retained molecules (TS > 70%), the abundance data were expressed as a percentage of the sum of the total ion current (TIC). The results were expressed as average ± standard deviations.

### 2.5. Minimum Inhibitory Concentration (MIC) and Minimum Bactericidal Concentration (MBC) of AgNPs and EOs

The MIC of AgNPs and EOs was determined by following the microdilution method according to the Clinical and Laboratory Standard Institute guidelines [36]. As found in the literature, EOs were initially diluted (75%) in dimethyl sulfoxide (DMSO, Merck, Milan, Italy) to allow two-fold dilution in Mueller–Hinton broth (Microbiol, Italy), reaching a linear oil gradient from 18.75% (*v*/*v*) to 0.04% (*v*/*v*). AgNPs were dissolved in distilled water to reach an initial concentration of 2.048 mg/mL and create a gradient from 512 µg/mL to 1 µg/mL. When combined with EOs, AgNPs were diluted in DMSO to maintain the same antimicrobial gradient. Before testing EOs and AgNPs against field strains, ATCC cultures were used (*S. aureus* ATCC 6358, *Escherichia coli* ATCC 25922, *Klebsiella pneumoniae* subsp. *ozaenae* ATCC 11296, *Micrococcus yunnanensis* ATCC 7468, *Enterococcus casseliflavus* ATCC 12755, *Providencia rettgeri* ATCC 9250, *Proteus vulgaris* ATCC 7829, *Streptococcus agalactiae* ATCC 13813, *Salmonella enterica* subsp. *enterica* serovar Enteritidis ATCC 25928, see Supplemental Table S1). DMSO alone (100% *v*/*v*) was tested to rule out its potential antibacterial activity. Positive and negative controls were inserted into each 96-well plate consisting of bac-teria without tested molecules and broth alone, respectively. After 24 h of incubation at 37 °C, the MIC was visually determined as the lowest concentration that inhibited bacterial growth.

The MBC was determined by inoculating the entire volume of the wells (200 µL) into tubes with sterile Mueller–Hinton broth and incubating at 37 °C for 24 h. The MBC was indicated as the lowest concentration capable of killing the bacteria in the broth. The lack of growth in the tubes was verified with a densitometer (BioSan Densitometer, Riga, Latvia) and compared with the positive and negative controls.

## 3. Results

### 3.1. Phenotypic and Molecular Profiling of Antibiotic Resistance

All *S. pseudintermedius* strains were found to be resistant to at least three antibiotic classes tested (*β*-lactams [B-LAC], Lincosamides [LIN], Tetracyclines [TET], Fluoroquinolones [FLQ], Aminoglycosides [AMN], Rifamycins [RIF], Macrolides [MAC]) and are therefore classified as MDR. Furthermore, all were found to be resistant to methicillin, which makes them MRSP (Table 1).

Resistance to penicillins and fluoroquinolones was 97.4% and 80.7%, respectively. The cephalosporins (all third-generation except for first-generation cephalexin) showed a slightly different trend, with a resistance of 89.5%. Interestingly, among the third-generation molecules tested, ceftriaxone showed the same resistance as cephalexin (78.9%), a first-generation cephalosporin. Penicillins were effective in about 2.6% of strains, while cephalosporins were effective in 6.6%. Lincosamides are the antibiotic category with the second-greatest resistance detected (around 95%). clindamycin, while only two strains were susceptible to the combination of lincomycin and spectinomycin. Approximately 47% of strains were susceptible to doxycycline. Fluoroquinolones are the second antibacterial category used in the treatment of pyoderma; among the three molecules, marbofloxacin is the most active (32%), followed by ENR (15.8%) and PRA (5%). Among aminoglycosides (which showed a sensitivity of 33%), amikacin was the molecule to which all strains were sensitive, followed by gentamicin and tobramycin with susceptibilities of 37% and 26%, respectively. In this study, rifampicin is the molecule to which all strains are susceptible, opposite of the macrolides trend (both AZM and E), which showed resistance to all *S. pseudintermedius* strains tested.

Genetically, all strains were positive for the *mecA* gene, conferring resistance to methicillin (and all penicillins and cephalosporins except ceftaroline), with 84% being positive for the *blaZ* gene, conferring resistance to *β*-lactams (in particular, ampicillin, amoxicillin, and amoxiclavulanate). The *aacA–aphD* gene (aminoglycoside resistance) was amplified in 78% of the bacteria, while the *tetM* and *tetK* genes were present in 5 and 7 strains, respectively.

**Figure 1:**
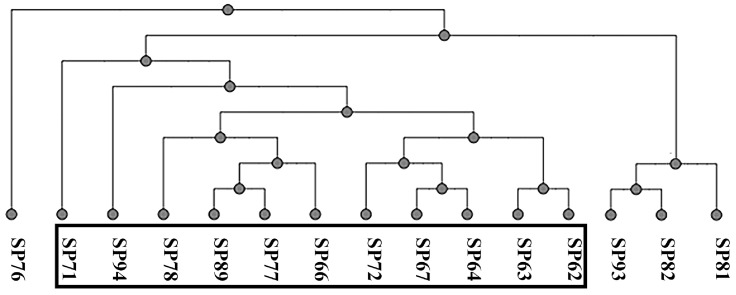
Pangenome-based UPGMA cluster analysis.

All the strains were full-length sequenced using a third-generation sequencing machine [41]. The Multi-locus Sequence Typing (MLST) analysis was conducted using an online tool (accessed on November 2023), and three different sequence types (STs) were found; ST71 was the most prevalent (11/15; 73%), followed by ST258 (3/15; 20%) and ST301 (1/15; 7%). Figure 1 represents the UPGMA cluster derived from the pangenome analysis with an online tool (accessed on November 2023). The black box includes the SP strains of ST71, which share the staphylococcal chromosome cassette (SCC)*mec* type II–III.

### 3.2. Characterization of Antimicrobial Molecules

#### 3.2.1. AgNPs

**Figure 2:**
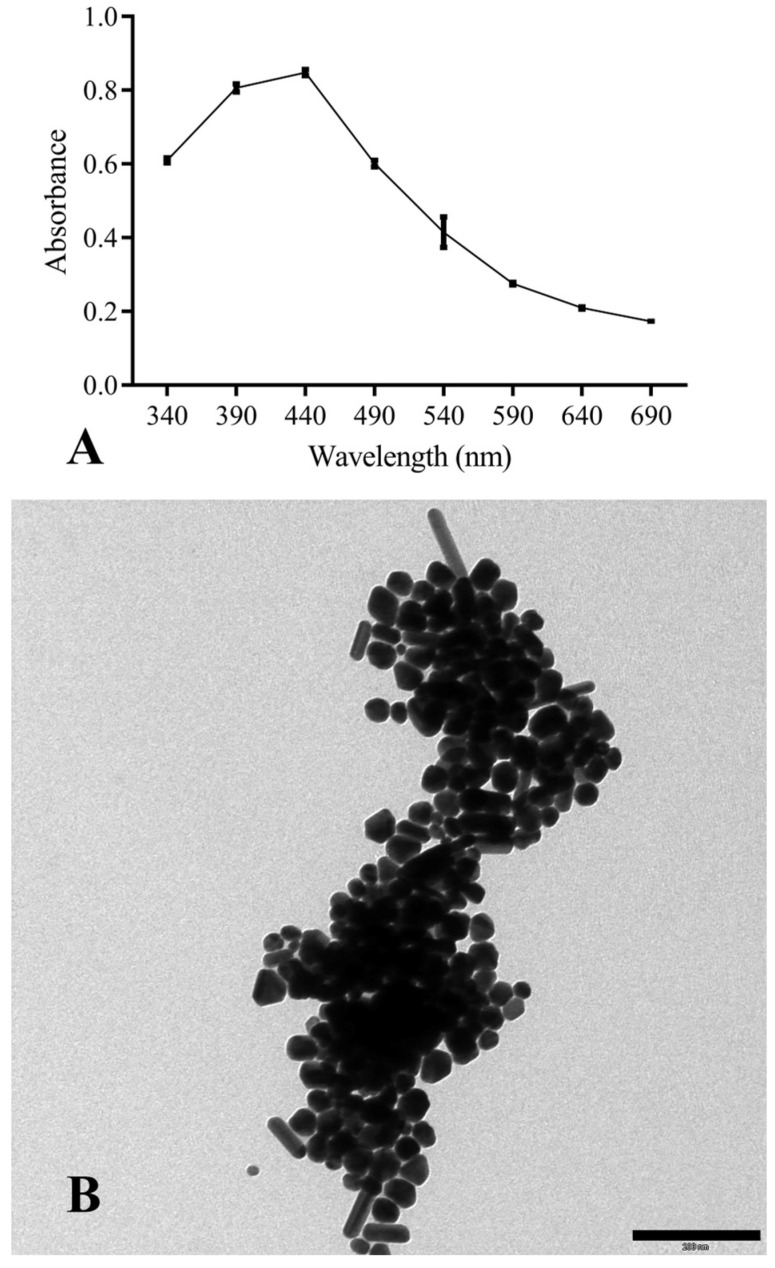
(**A**) UV-Vis absorption spectra of chemically-synthesized AgNPs; (**B**) TEM morphological analysis of NPs (scale bar corresponds to 200 nm).

AgNPs were obtained by chemical synthesis from silver nitrate as the Ag^+^ donor and TSC as the reducing and stabilizing agent. The first macroscopic characterization indicative of the reduction of Ag^+^ to Ag^0^ was the observation (over time) of the solution’s color change, which progressively turned from transparent to dark brown. Once the synthesis was completed, ultraviolet-visible spectroscopy (UV-Vis spectroscopy) confirmed the presence of metal nanoparticles. This technique makes it possible to derive qualitative–quantitative information by exploiting the ability of different substances to absorb a given wavelength, which in the case of AgNPs is 440 nm (Figure 2A).

During the synthesis, the pH of the reaction was not controlled; consequently, the morphology of the particles (Figure 2B) was not homogeneous but had different symmetries: triangular, pentagonal, hexagonal, filiform, spherical, and cubic.

The size analysis was performed with ImageJ2 software (v. 1.54h), which allowed the area of NPs with spherical symmetry to be derived (for the other symmetries, although it was possible to determine the area, the volume could not be calculated). The synthesized AgNPs had a size of 15 ± 2.7 nm. A factor affecting their antibacterial capacity is the surface area/volume ratio (S/V), which is linked to the contact surface area between the particles and the pathogen. In this work, the S/V ratio was 0.56 ± 0.09 nm^2^/nm^3^.

#### 3.2.2. EOs

The constituents of the four EOs are reported in Table 2, listed in ascending order of their linear retention indices (LRIs) on the apolar column. A total of 124 compounds belonging to different chemical classes have been identified. Of these, 35 were detected in AR, representing 96.68% of EO, 75 in AB (98.09%), 77 in GI (91.75%), and 57 in RO (98.65%). In all EOs, the monoterpenic fraction (40.55%, 39.68%, 39.56%, and 60.17%, respectively) prevailed over the sesquiterpenic fraction (6.96%, 23.54%, 30.64%, and 17.73%). Oxygenated monoterpenes were more abundant than monoterpene hydrocarbons in AR (22:1), lower in AB (1:2) and GI (1:1.2), and in similar percentages in RO (1:1). In detail, cis- or trans-*p*-menth-2-en-1-ol characterized AR and AB (36.14% and 6.1%, respectively), GI (5.96%) together with 1,6-octadien-3-ol, 3,7-dimethyl- (6.12%), and RO (7.22%) together with cis-*β*-terpineol (8.1%). Among the sesquiterpenoids, oxygenated sesquiterpenes were the main components in AR, AB, and GI (6.62%, 12.57%, and 21.98%) with *α*-sinensal (1.91%), *α*-santalene (3.71%), and guaiol (3.98%) as the major compounds, respectively. Sesquiterpene hydrocarbons predominated in RO (12.41%), and (+)-sativene was the most abundant (5.71%). Diterpenoids were present in very low percentages (0.55% to 3.58%) in three of the four samples, and absent in RO.

### 3.3. Antibacterial Activity of EOs and NPs

In this work, the antibacterial action of AgNPs and EOs of Spruce (AB), Orange (AR), Juniper (GI), and Rosemary (RO) was investigated by determining the MIC and MBC (Table 3 and Table 4). In all the tests performed, the viability control with DMSO alone (100% *v*/*v*) showed that this substance (used to make the oils soluble and disperse the nanoparticles) did not interfere with the biological action observed by the tested molecules.

The results reported in Table 3 and Table 4 show that both AgNPs and EOs succeed in inhibiting and killing *S. pseudintermedius* strains. With 18/19 (94.73%) strains exhibiting MIC values greater than 1/128 *v*/*v*, AB produced the greatest inhibitory effects against all strains of *S. pseudintermedius* among the investigated essential oils. This was followed by GI (17/19; 89.47%), AR (9/19; 47.36%), and RO (9/17; 52.94%), respectively. AB had the highest level of bactericidal activity, as it was able to eliminate 13/18 (72.2%) strains with an EO concentration that was more than 1/32 *v*/*v*. This was followed by GI (13/19; %), AR (6/18; 33.3%), and RO (1/17; 5.8%), depending on the strain.

When AgNPs were introduced to EOs, the same pattern that was seen for EOs that were evaluated on their own was discovered. Following the incorporation of NPs, a decrease in the values of both the MIC and MBC was found for each and every EOs that was examined. More specifically, the MIC values for AB ranged from 1:256 to 1:2048 *v*/*v*, which led to the inhibition of 17/19 (89.4%) *S. pseudintermedius* strains at concentrations equal to or higher than 1:1024. This was followed by the inhibition of GI (15/19; 78.9%), AR (13/19; 68.4%), and RO (13/19; 78.9%), respectively. A distinct pattern was seen in the bactericidal efficacy of the combination when compared to the minimum inhibitory concentration. To be more specific, 17/19 (89.4%) of the strains that were examined were eliminated by AR oil at a concentration that was equal to or greater than 1:512. This was followed by GI (16/19; 84.2%), AB (14/19; 73.68%), and RO (12/19; 63.1%), in that order.

## 4. Discussion

The global increase in the prevalence of MDR, and more broadly, MRSP strains isolated from both cases of canine pyoderma and human infections, is of growing interest in the public health landscape. If this fact is also associated with the limited pharmacological choice resulting from the lack of new antibiotic molecules, developing alternatives to classic antimicrobial therapy must be considered. Within the realm of novel therapeutic possibilities, the rediscovery of alternative treatments based on the use of natural ingredients and the creation and characterization of nanotechnologies are piquing the scientific community’s attention. 

The antibiogram findings suggest a scenario of widespread resistance, making it difficult for the veterinarian to select the optimal antibiotic to administer. Because all strains were *mecA*-positive, no *β*-lactam antibiotics may be used to treat infection with this disease. The only two compounds all strains were susceptible to were amikacin (aminoglycoside) and rifampicin (rifamycins), both nephrotoxic and hepatotoxic. The resistance profile found in this work is consistent with that obtained in another Italian investigation that examined MRSP and MSSP strains isolated from dogs in Milan and Naples [42].

The role of EOs against the most common pathogens has been investigated extensively [43,44,45,46,47,48,49]. On the other hand, data on their efficacy against *S. pseudintermedius* are scarce. The results of this work are quite promising and confirmed those of the limited available literature. Indeed, in different studies, the MIC value of RO towards *S. pseudintermedius* was 0.5% [50]. For *S. aureus*, multiple results are present: 1.5% [51]; 1.5–3.6% [52]; 2% [53]. Fu et al. found an MIC value for *S. aureus* and *S. epidermidis* of 0.125% and 0.250%, respectively [54]. No data from similar studies using the same pathogen are available for other EOs used in this work. Only one Italian study (Nocera et al., 2020) demonstrated that MRSP strains isolated from dogs are susceptible to the action of some EOs, albeit different ones [55]. As regards the main constituents of the four EOs tested, although their specific activity against SP has not yet been documented, some of them, such as *p*-menthenols, have already shown a significant antimicrobial potential against some pathogenic strains [56] or have been recognized (e.g., 1,6-octadien-3-ol, 3,7-dimethyl-, cis-*β*-terpineol, *α*-santalene, or guaiol) as responsible for the antibacterial activity of the EOs in which they were present in greater quantities [57,58,59,60]. However, it is now well-established that minor active compounds coexisting in essential oils can contribute to their registered antimicrobial activity [61]

In the present study, NPs were synthesized using a chemical method, which allowed the obtaining of particles of different symmetries. This leads to the second limitation found in this work that concerns the lack of homogeneity in the morphology of the particles obtained, as described in the results (and confirmed by other authors). The failure to control the pH of the reaction favored the synthesis of multiple morphologies which, nevertheless, exerted a bactericidal action certainly superior to one-dimensional colloidal solutions. This approach is certainly not free from potential errors, but it offers better results when compared to plant- or bacteria-mediated biological synthesis [5].

## 5. Conclusions

Our investigation has discovered, for the first time and for the best of our knowledge, the antibacterial effects of our chosen essential oils against methicillin-resistant *Staphylococcus pseudintermedius* strains obtained from dogs with pyoderma. Among the EOs tested, AR showed the most promising results both tested alone and in combination with AgNPs.

## Figures and Tables

**Table 1:** Antibiotic resistance profiles of the *S. pseudintermedius* strains included in the study.

Antibiotics																				R (%)	I (%)	S (%)
OX_5_	R	R	R	S	R	R	R	R	R	R	R	S	R	R	S	R	R	R	R	100.0	0	0.0
AMC_30_	R	R	R	R	R	R	R	R	R	R	R	S	R	R	S	R	R	R	R	89.5	0	10.5
AMO_30_	R	R	R	R	R	R	R	R	R	R	R	R	R	R	R	R	R	R	R	100	0	0
CAR_100_	R	R	R	R	R	R	R	R	R	R	R	R	R	R	R	R	R	R	R	100	0	0
CL_30_	R	R	R	R	R	R	R	R	R	R	R	S	R	R	S	S	R	S	R	78.9	0	21.1
CVN_30_	R	R	R	R	R	R	R	R	R	R	R	R	R	R	R	R	R	R	R	100	0	0
EFT_30_	R	R	R	R	R	R	R	R	R	R	R	R	R	R	R	R	R	R	R	100	0	0
CRO_30_	R	R	R	S	R	R	R	R	R	R	R	I	R	R	I	I	R	R	R	78.9	15.8	5.3
DA_10_	R	R	R	R	R	R	R	R	R	R	R	R	R	R	R	R	R	R	R	100	0	0
LC-SP_15_	R	I	R	R	R	R	I	R	R	R	R	R	R	R	R	R	R	R	R	89.5	10.5	0
DO_30_	S	R	R	S	R	R	R	I	S	S	S	R	R	R	R	S	S	S	S	47.4	5.3	47.4
ENR_5_	R	R	R	R	R	R	R	R	R	R	R	S	R	R	S	S	R	I	R	78.9	5.3	15.8
MAR_5_	R	S	R	S	R	R	R	R	R	R	R	S	R	R	S	S	R	S	R	68.4	0	31.6
PRA_5_	R	R	R	R	R	R	R	R	R	R	R	R	R	R	S	R	R	R	R	94.7	0	5.3
AK_30_	S	S	S	S	S	S	S	S	S	S	S	S	S	S	S	S	S	S	S	0	0	100
CN_30_	R	R	S	I	I	S	S	S	R	I	R	S	R	R	R	S	R	S	R	47.4	15.8	36.8
N_30_	R	R	R	R	I	I	R	R	R	I	R	I	R	R	R	R	R	R	R	78.9	21.1	0
K_30_	R	R	R	R	R	R	R	R	R	R	R	R	R	R	R	R	R	R	R	100	0	0
TOB_10_	R	I	R	R	S	S	R	R	R	R	R	S	R	R	R	S	R	S	R	68.4	5.3	26.3
RD_30_	S	S	S	S	S	S	S	S	S	S	S	S	S	S	S	S	S	S	S	0	0	100
AZM_15_	R	R	R	R	R	R	R	R	R	R	R	R	R	R	R	R	R	R	R	100	0	0
E_30_	R	R	R	R	R	R	R	R	R	R	R	R	R	R	R	R	R	R	R	100	0	0

Abbreviations: R = Resistant; I = Intermediate; S = Susceptible; for antibiotic abbreviations, refer to Section 2.1. “Determination of antimicrobial profiles”.

**Table 2:** Chemical composition (%) of *Citrus sinensis* (AR), *Abies alba* (AB), *Juniperus communis* (GI), and *Rosmarinus officinalis* (RO) essential oils determined by GC/MS.

# ^1^		TS ^2^	LRI ^3^	AR ^4^	AB ^5^	GI ^6^	RO ^7^
				% TIC			
1	α-Sinensal	80.2	855	1.91 ± 0.06	tr	-	-
2	Ethanol, 2,2′-oxybis-	87.7	967	11.0 ± 5.01	5.08 ± 4.67	5.19 ± 0.35	8.25 ± 0.17
3	Methyl DL Leucate	82.2	968	3.01 ± 1.14	2.63 ± 1.38	2.14 ± 1.09	2.73 ± 0.84
4	Acetic acid, 2-ethylbutyl ester	82.9	976	0.28 ± 0.05	tr	tr	tr
5	Pseudolimonene	91.0	994	0.17 ± 0.04	2.92 ± 5.43	4.10 ± 5.59	6.54 ± 4.69
6	Nonane, 3-methyl-	74.2	995	0.11 ± 0.02	-	tr	tr
7	α-Phellandrene	94.8	996	0.22 ± 0.04	tr	4.24 ± 5.53	0.37 ± 0.29
8	3-Butenoic acid, 3-methyl-, trimethylsilyl ester	80.0	1004	2.35 ± 0.55	0.18 ± 0.33	0.34 ± 0.17	0.26 ± 0.27
9	α-Terpinene	93.0	1008	tr	8.16 ± 7.78	0.82 ± 2.09	5.21 ± 4.19
10	4-Carene	93.8	1009	tr	5.87 ± 5.13	1.50 ± 1.99	2.76 ± 3.69
11	2-Carene	93.6	1009	tr	3.57 ± 4.19	1.03 ± 1.43	2.67 ± 0.20
12	*α*-Tesabinenene	91.2	1014	tr	8.16 ± 7.78	0.82 ± 2.09	5.21 ± 4.19
13	*β*-cis-Ocimene	94.4	1022	0.63 ± 0.24	2.94 ± 5.14	0.30 ± 0.80	5.54 ± 7.41
14	3-Carene	93.2	1029	tr	tr	tr	0.58 ± 0.67
15	*β*-Terpinene	92.8	1036	tr	3.19 ± 4.80	0.27 ± 0.72	5.81 ± 7.58
16	Crithmene	94.6	1054	0.74 ± 0.30	tr	2.55 ± 3.69	0.30 ± 0.44
17	Benzene, butyl-	85.4	1056	tr	0.69 ± 0.60	0.95 ± 1.32	0.19 ± 0.26
18	4-Thujanol	92.1	1065	0.47 ± 0.02	-	0.15 ± 0.22	-
19	Terpinolene	95.5	1080	tr	0.10 ± 0.09	5.90 ± 8.21	0.13 ± 0.18
20	1,6-Octadien-3-ol, 3,7-dimethyl-	85.1	1094	-	0.32 ± 0.30	6.12 ± 8.51	2.00 ± 2.61
21	Benzene, 1-ethyl-2,3-dimethyl-	92.0	1096	tr	0.14 ± 0.14	0.35 ± 0.87	tr
22	Benzene, 1,2,4,5-tetramethyl-	89.8	1101	tr	0.15 ± 0.14	0.40 ± 1.01	tr
23	5,6-dehydrocamphor	81.6	1104	0.22 ± 0.09	0.15 ± 0.27	3.19 ± 8.42	0.10 ± 0.14
24	*p*-Mentha-1,3,8-triene	92.3	1106	tr	tr	0.37 ± 1.03	tr
25	*Allo*-Ocimene	88.0	1113	tr	0.24 ± 0.44	0.15 ± 0.21	0.37 ± 0.57
26	Disulfide, methyl (methylthio)methyl	72.4	1119	4.12 ± 0.49	tr	tr	-
27	(Z)-*p*-Menthen-2-en-1-ol	90.8	1125	36.14 ± 0.43	0.15 ± 0.25	0.41 ± 1.06	7.22 ± 5.89
28	Terpineol, cis-*β*-	87.8	1133	0.98 ± 0.01	0.25 ± 0.23	0.11 ± 0.14	8.10 ± 6.36
29	(E)-*p*-Menthen-2-en-1-ol	93.5	1137	tr	6.10 ± 7.95	5.96 ± 8.29	3.67 ± 0.62
30	Benzene, 1-ethenyl-4-methoxy-	86.8	1158	tr	tr	0.20 ± 0.44	0.36 ± 0.44
31	Ethanone, 1-(2-methylphenyl)-	91.8	1176	tr	0.33 ± 0.64	0.59 ± 1.59	5.47 ± 8.30
32	*γ*-Terpineol	90.4	1182	tr	tr	tr	0.18 ± 0.23
33	Isopentyloxyethyl acetate	82.1	1184	tr	0.26 ± 0.49	tr	tr
34	*α*-Terpineol	88.7	1191	tr	-	0.19 ± 0.46	0.49 ± 0.43
35	(L)-*α*-Terpineol	88.6	1191	tr	-	1.66 ± 3.94	0.40 ± 0.26
36	Ethanone, 1-(3-methylphenyl)-	92.9	1192	tr	0.31 ± 0.60	0.73 ± 1.58	tr
37	Phenol, 2,4,6-trimethyl-	90.7	1202	tr	-	1.61 ± 4.47	0.20 ± 0.31
38	(1S,4S)-Dihydrocarvone	84.0	1202	tr	0.24 ± 0.46	tr	-
39	Undecanal	82.6	1290	tr	-	0.11 ± 0.29	-
40	Citronellyl Acetate	84.5	1336	tr	0.10 ± 0.09	tr	tr
41	2,3-Dimethyldodecane	80.7	1346	tr	tr	0.10 ± 0.19	0.20 ± 0.32
42	(E,Z)-jasmone	81.4	1349	tr	tr	0.29 ± 0.62	tr
43	Tridecane, 7-methyl-	83.3	1353	tr	tr	0.19 ± 0.19	0.23 ± 0.34
44	*α*-Longipinene	95.6	1360	0.21 ± 0.05	tr	0.44 ± 1.20	tr
45	Pentanoic acid, heptyl ester	86.0	1364	tr	tr	0.14 ± 0.40	tr
46	Cyclosativene	93.2	1367	0.13 ± 0.00	0.83 ± 1.60	0.49 ± 1.36	tr
47	2-Octenal, 2-butyl	87.7	1367	1.60 ± 0.05	0.20 ± 0.35	0.15 ± 0.23	0.11 ± 0.15
48	Copaene	86.6	1367	tr	0.17 ± 0.24	0.58 ± 1.59	tr
49	*α*-Cubebene	97.8	1374	tr	0.90 ± 1.71	0.48 ± 1.29	tr
50	Di-epi-α-cedrene	95.9	1384	tr	0.88 ± 1.56	0.18 ± 0.49	tr
51	α-Cedrene	95.6	1384	tr	tr	0.19 ± 0.53	0.47 ± 0.76
52	*β*-Cubebene	95.9	1394	tr	tr	0.22 ± 0.59	0.56 ± 0.74
53	Acetic acid, decyl ester	90.8	1397	0.42 ± 0.02	1.93 ± 3.45	tr	0.40 ± 0.61
54	2H-Pyran-2-one, 6-pentyl-	84.9	1408	tr	2.96 ± 5.53	tr	2.74 ± 4.38
55	*β*-Patchoulene	90.8	1408	tr	0.14 ± 0.17	0.20 ± 0.50	tr
56	Terpinyl propionate	87.5	1418	tr	2.67 ± 5.10	tr	-
57	Lynalyl butyrate	90.3	1429	tr	tr	tr	0.84 ± 1.28
58	*β*-Gurjunene	95.4	1429	tr	tr	0.44 ± 1.20	tr
59	*β*-Santalene	84.1	1432	tr	0.32 ± 0.39	tr	tr
60	*β*-Selinene	86.5	1432	tr	0.37 ± 0.64	tr	-
61	*α*-Caryophyllene	91.0	1443	tr	0.24 ± 0.22	tr	0.13 ± 0.17
62	Spathulenol	89.6	1444	tr	tr	0.46 ± 1.26	tr
63	α Himachalene	94.4	1450	tr	0.50 ± 0.66	-	tr
64	Acetophenone, 4′-hydroxy-	79.5	1453	tr	0.41 ± 0.72	-	tr
65	*β*-Humulene	92.9	1458	tr	0.52 ± 0.71	tr	tr
66	α-Santalene	87.8	1459	0.42 ± 0.1	3.71 ± 5.86	0.16 ± 0.16	0.97 ± 1.47
67	11-Dodecenol	81.9	1461	tr	0.56 ± 0.74	tr	0.13 ± 0.21
68	γ-Gurjunene	86.1	1470	tr	0.39 ± 0.73	tr	0.12 ± 0.12
69	Butanoic acid, 3-methyl-, 1-ethenyl-1,5-dimethyl-4-hexenyl ester	87.5	1471	20.78 ± 0.66	3.74 ± 6.58	0.25 ± 0.40	1.59 ± 1.32
70	(E)-Isoeugenol	82.3	1474	tr	0.15 ± 0.19	0.12 ± 0.29	0.12 ± 0.18
71	*β*-Chamigrene	95.1	1475	tr	0.20 ± 0.29	0.10 ± 0.27	1.42 ± 2.23
72	10-Dodecenol	85.2	1479	tr	1.02 ± 1.74	tr	0.24 ± 0.34
73	*β*-Guaiene	88.5	1484	tr	0.25 ± 0.43	0.14 ± 0.25	1.52 ± 1.96
74	γ-Cadinene	85.8	1488	tr	tr	0.14 ± 0.24	0.66 ± 0.99
75	α-Bisabolene	85.1	1494	tr	0.76 ± 1.00	tr	0.64 ± 1.01
76	Valencene (isomer R)	89.1	1495	tr	0.33 ± 0.38	tr	0.56 ± 0.84
77	Valencene (isomer S)	94.4	1497	tr	0.77 ± 0.81	tr	tr
78	*β*-Bisabolene	83.2	1500	tr	0.64 ± 1.18	tr	tr
79	2,4-Dodecadienal, (E,E)-	79.8	1502	0.34 ± 0.00	0.26 ± 0.37	tr	tr
80	(Z,E)-α-Farnesene	82.4	1505	tr	0.52 ± 0.49	0.41 ± 1.08	tr
81	α-Muurolene	91.3	1508	tr	tr	2.09 ± 5.77	tr
82	δ-Guaiene	88.5	1508	tr	0.14 ± 0.19	tr	tr
83	Epizonarene	82.1	1522	tr	tr	2.21 ± 6.14	tr
84	Sesquiphellandrene	84.4	1522	0.21 ± 0.04	0.80 ± 1.34	0.12 ± 0.32	tr
85	(+)-Sativene	85.6	1523	tr	1.63 ± 2.51	-	5.71 ± 9.07
86	Hedycaryol	85.8	1528	tr	tr	tr	0.56 ± 0.88
87	Isocadiene	95.2	1534	tr	tr	2.72 ± 7.53	tr
88	Butanoic acid, 3,7-dimethyl-6-octenyl ester	90.9	1536	tr	1.08 ± 1.07	tr	0.14 ± 0.08
89	Eudesma-3,7(11)-diene	89.6	1538	tr	tr	2.83 ± 7.41	tr
90	*β*-Himachalene	73.0	1561	tr	0.12 ± 0.19	0.58 ± 0.79	0.25 ± 0.38
91	Nerolidol	83.7	1567	tr	0.28 ± 0.51	tr	2.00 ± 3.16
92	Caryophyllene oxide	87.8	1576	tr	0.36 ± 0.58	0.16 ± 0.20	tr
93	*β*-Elemenone	81.2	1578	tr	0.51 ± 0.91	0.22 ± 0.48	tr
94	Carotol	85.9	1583	-	0.11 ± 0.11	1.13 ± 3.16	tr
95	Boronia butenal	84.4	1586	tr	2.63 ± 4.99	tr	tr
96	Germacrene B	89.0	1589	tr	0.28 ± 0.25	0.20 ± 0.31	tr
97	Dodecan-1-yl acetate	90.3	1590	0.39 ± 0.01	0.11 ± 0.10	0.20 ± 0.35	tr
98	Guaiol	88.4	1597	tr	0.11 ± 0.10	3.98 ± 9.16	0.10 ± 0.07
99	Cedrenol	85.0	1607	1.17 ± 0.55	1.19 ± 1.76	0.27 ± 0.72	tr
100	α-Eudesmol	79.0	1607	tr	0.14 ± 0.14	0.28 ± 0.76	tr
101	Cubenol	75.5	1611	0.20 ± 0.01	0.48 ± 0.29	0.20 ± 0.54	0.24 ± 0.34
102	Hinesol	85.0	1620	tr	0.37 ± 0.58	3.55 ± 9.82	0.53 ± 0.83
103	Ledol	81.3	1620	0.30 ± 0.01	0.45 ± 0.42	0.52 ± 1.44	tr
104	γ-Eudesmol	88.4	1625	0.10 ± 0.00	tr	3.88 ± 5.41	tr
105	*β*-Homocyclocitral	77.5	1627	-	0.31 ± 0.47	-	-
106	τ-Cadinol	92.7	1642	tr	0.32 ± 0.60	3.67 ± 9.02	tr
107	Geranyl valerate	79.7	1648	2.49 ± 0.09	tr	tr	tr
108	Blumenol C	70.4	1673	tr	1.60 ± 3.05	tr	tr
109	2(1H)-Quinolinone, 1-methyl-	82.0	1673	tr	tr	3.53 ± 9.77	0.19 ± 0.30
110	δ-Cadinol	82.3	1678	tr	tr	0.54 ± 0.87	-
111	Phenol, 3-methyl-5-(1-methylethyl)-, methylcarbamate	90.7 83.6	1693	tr	0.72 ± 1.17	tr	0.17 ± 0.27
112	Cedren-13-ol, 8-	84.8	1692	0.22 ± 0.01	0.22 ± 0.40	tr	-
113	(Z,E) Farnesyl acetate	83.5	1699	1.04 ± 0.23	0.51 ± 0.50	tr	tr
114	1-Heptadecene	71.8	1711	tr	0.18 ± 0.22	tr	tr
115	(E,E) Farnesal	83.4	1718	tr	0.32 ± 0.59	-	-
116	Solavetivone	73.4	1817	tr	0.18 ± 0.34	-	tr
117	(E,E) Farnesyl acetate	85.4	1833	tr	1.11 ± 2.09	-	tr
118	Rimuene	82.3	1905	tr	tr	0.35 ± 0.93	-
119	Kaur-16-ene, (8-*β*,13-*β*)-	82.3	2015	tr	0.15 ± 0.19	tr	tr
120	Epimanool	79.6	2019	2.25 ± 0.07	tr	0.10 ± 0.27	tr
121	(E,E) Farnesyl lactone	86.8	1920	0.21 ± 0.08	-	tr	-
122	Farnesylacetone	85.0	1926	0.29 ± 0.07	1.70 ± 3.26	-	tr
123	Geranyllinalool	89.2	2025	tr	0.38 ± 0.44	tr	tr
124	Kaur-16-ene	86.7	2046	1.56 ± 0.34	1.03 ± 1.79	0.10 ± 0.27	tr
	Class of components						
	Monoterpenoids			40.55	39.68	39.56	60.17
	Monoterpene hydrocarbons			1.76	26.92	21.66	29.91
	Oxygenated monoterpenes			38.79	12.76	17.90	30.26
	Diterpenoids			3.78	1.56	0.55	-
	Diterpene hydrocarbons			1.53	1.18	0.45	-
	Oxygenated diterpenes			2.25	0.38	0.10	-
	Sesquiterpenoids			6.96	23.54	30.64	17.73
	Sesquiterpene hydrocarbons			0.34	10.97	8.66	12.41
	Oxygenated sesquiterpenes			6.62	12.57	21.98	5.32
	Others			45.39	33.31	21.00	20.75
				96.68	98.09	91.75	98.65

^1^ The components are reported according to their elution order on apolar column; ^2^ Total Score (Tsugawa et al., 2015); ^3^ Linear Retention indices measured on apolar column; ^4^ Percentage mean values of AR components; ^5^ Percentage mean values of AB components; ^6^ Percentage mean values of GI components; ^7^ Percentage mean values of RO components; Abbreviations: - = Not detected; tr = traces (mean value < 0.1% TIC).

**Table 3:** Minimum inhibitory concentration (MIC) of the tested EOs and NPs against *S. pseudintermedius* strains.

Strains	NPs	AB	AB:NPs	AR	AR:NPs	GI	GI:NPs	RO	RO:NPs
35	1:128	1:128	1:1024	1:256	1:512	1:256	1:512	1:64	1:512
39	1:256	1:128	1:1024	1:128	1:512	1:256	1:512	na	1:512
45	1:256	1:64	1:1024	1:32	1:1024	1:128	1:1024	1:64	1:1024
46	1:256	1:512	1:2048	1:128	1:1024	1:32	1:1024	na	1:1024
62	1:256	1:2048	1:2048	1:64	1:2048	1:512	1:1024	1:256	1:1024
63	1:512	1:2048	1:2048	1:64	1:2048	1:512	1:1024	1:128	1:1024
64	1:256	1:512	1:1024	1:64	1:1024	1:128	1:2048	1:64	1:1024
66	1:256	1:2048	1:2048	1:64	1:2048	1:256	1:2048	1:128	1:512
67	1:128	1:1024	1:1024	1:256	1:1024	1:256	1:1024	1:128	1:1024
71	1:128	1:2048	1:2048	1:512	1:1024	1:128	1:1024	1:64	1:1024
72	1:256	1:2048	1:512	1:128	1:512	1:128	1:512	1:128	1:1024
76	1:128	1:128	1:256	1:2048	1:1024	1:256	1:1024	1:128	1:256
77	1:128	1:512	1:1024	1:32	1:512	1:512	1:512	1:256	1:256
78	1:128	1:256	1:2048	1:64	1:1024	1:256	1:1024	1:64	1:256
81	1:256	1:1024	1:1024	1:128	1:512	1:512	1:1024	1:256	1:1024
82	1:128	1:1024	1:1024	1:64	1:1024	1:256	1:1024	1:256	1:1024
89	1:256	1:1024	1:1024	1:128	1:1024	1:128	1:2048	1:64	1:1024
93	1:256	1:1024	1:2048	1:64	1:2048	1:128	1:1024	1:64	1:1024
94	1:256	1:512	1:1024	1:64	1:512	1:64	1:1024	1:64	1:1024

Abbreviation: na = not available.

**Table 4:** Minimum bactericidal concentration (MBC) of the tested EOs and NPs against *S. pseudintermedius* strains.

Strains	NPs	AB	AB:NPs	AR	AR:NPs	GI	GI:NPs	RO	RO:NPs
35	1:4	1:4	1:256	1:4	1:256	1:4	1:128	1:4	1:128
39	1:8	1:8	1:256	1:128	1:256	1:64	1:256	na	1:256
45	1:4	1:8	1:2048	na	1:1024	1:4	1:512	1:4	1:512
46	1:8	1:16	1:2048	1:4	1:1024	1:4	1:512	na	1:512
62	1:4	1:64	1:2048	1:32	1:512	1:64	1:512	1:4	1:512
63	1:8	na	1:1024	1:32	1:2048	1:128	1:1024	1:16	1:512
64	1:8	1:32	1:512	1:4	1:1024	1:16	1:2048	1:8	1:512
66	1:4	1:32	1:2048	1:8	1:2048	1:32	1:2048	1:8	1:512
67	1:32	1:512	1:1024	1:128	1:1024	1:16	1:1024	1:8	1:512
71	1:32	1:128	1:1024	1:16	1:1024	1:32	1:1024	1:4	1:1024
72	1:16	1:32	1:256	1:16	1:512	1:32	1:512	1:8	1:1024
76	1:16	1:1024	1:256	1:64	1:1024	1:64	1:512	1:8	1:128
77	1:8	1:32	1:1024	1:8	1:512	1:32	1:512	1:8	1:256
78	1:16	1:128	1:512	1:8	1:512	1:32	1:512	1:8	1:256
81	1:64	1:512	1:1024	1:64	1:1024	1:128	1:1024	1:32	1:512
82	1:32	1:128	1:256	1:16	1:512	1:32	1:512	1:16	1:1024
89	1:4	1:8	1:2048	1:4	1:512	1:16	1:512	1:8	1:512
93	1:8	1:64	1:1024	1:8	1:512	1:32	1:512	1:16	1:256
94	1:8	1:32	1:1024	1:4	1:512	1:32	1:256	1:8	1:256

Abbreviation: na = not available.

## Data Availability

All data presented in this study are available from the corresponding author upon request.

## Supplementary Materials

The following supporting information can be downloaded at: https://www.mdpi.com/article/10.3390/pathogens13020156/s1, Table S1: Minimum inhibitory concentration (MIC) and minimum bactericidal concentration (MBC) of the tested EOs against bacterial reference strains.
