# Supplementary material for: Antibacterial Potential of Essential Oils and Silver Nanoparticles against Multidrug-Resistant Staphylococcus pseudintermedius Isolates
Source: Pathogens. 2024 Feb 9;13(2):156. doi: 10.3390/pathogens13020156 (PMC10893185; doi:10.3390/pathogens13020156)
Supplement: Supplementary file 1 [file pathogens-13-00156-s001.zip › pathogens-2761084-supplementary.pdf]

Supplementary Table S1. Minimum inhibitory concentration (MIC) and minimum bactericidal concentration (MBC) of the tested EOs against bacterial reference strains

MIC	ECO	SAU	KPN	MIC	ECA	PRE	PVU	SAG	SAL
AB	1:64	1:512	1:512	1:1024	1:128	1:2048	1:2048	1:2048	1:64
RO	1:128	1:512	1:256	1:512	1:256	1:2048	1:2048	1:1024	1:256
GI	1:64	1:2048	1:1024	1:256	1:128	1:256	>1:4	1:1024	1:64
AR	1:32	1:8	1:8	1:32	1:1024	1:256	1:2048	1:2048	1:128
NPs	1:128	1:512	1:512	1:512	1:512	1:256	1:256	1:256	1:128
MBC	ECO	SAU	KPN	MIC	ECA	PRE	PVU	SAG	SAL
AB	1:8	1:128	1:128	1:128	1:4	1:64	1:1024	1:1024	1:4
RO	1:64	1:512	1:64	1:64	1:8	1:2048	1:2048	1:512	1:128
GI	1:8	1:8	1:256	1:64	1:64	1:64	>1:4	1:128	1:16
AR	1:4	1:4	1:4	1:4	1:4	1:32	1:1024	1:1024	1:16
NPs	1:32	1:128	1:128	1:128	1:128	1:256	1:128	1:256	1:32

Abbreviations: ECO= *Escherichia coli* ATCC 25922, SAU= *S. aureus* ATCC 6358, KPN= *Klebsiella pneumoniae* subsp. *ozaenae* ATCC 11296, MIC= *Micrococcus yunnanensis* ATCC 7468, ECA= *Enterococcus casseliflavus* ATCC 12755, PRO= *Providencia rettgeri* ATCC 9250, PVU= *Proteus vulgaris* ATCC 7829, SAG= *Streptococcus agalactiae* ATCC 13813, SAL= *Salmonella enterica* subsp. *enterica* serovar Enteritidis ATCC 25928
